# Social Media Screening and Procedural Justice: Towards Fairer Use of Social Media in Selection

**DOI:** 10.1007/s10672-021-09372-4

**Published:** 2021-04-19

**Authors:** Eva Vosen

**Affiliations:** Faculty of Economics and Business Administration, Catholic University of Eichstätt-Ingolstadt, Auf der Schanz 49, 85049 Ingolstadt, Germany

**Keywords:** Social media, Social media screening, Social media assessment, Selection, Procedural justice

## Abstract

Companies have started using social media for screening applicants in the selection process. Thereby, they enter a low-cost source of information on applicants, which potentially allows them to hire the right person on the job and avoid irresponsible employee behaviour and negligent hiring lawsuits. However, a number of ethical issues are associated with this practice, which give rise to the question of the fairness of social media screening. This article aims to provide an assessment of the procedural justice of social media screening and to articulate recommendations for a fairer use of social media in the selection process. To achieve this, a systematic literature review of research articles pertaining to social media screening has been conducted. Thereby, the benefits and ethical issues relating to social media screening, as well as recommendations for its use have been extracted and discussed against Leventhal’s (1980) rules of procedural justice. It turns out that without clear guidelines for recruiters, social media screening cannot be considered procedurally fair, as it opens up way too many opportunities for infringements on privacy, unfair discrimination, and adverse selection based on inaccurate information. However, it is possible to enhance the fairness of this practice by establishing clear policies and procedures to standardize the process.

## Introduction

The recent COVID-19 crisis has demonstrated the impact of digital technologies on the way work is organized: With the use of technology, work can to a large extent be done remotely and in isolation (Bapuji et al. 2020). For the first time, a great number of employees have found themselves working from home, and many managers have started leading remote teams (Caligiuri et al. 2020). These changes in work organization are expected to continue to affect the future of work and the role of technology in it (Bapuji et al. 2020). During the pandemic, firms with a high degree of digitalization have been able to effectively respond to the challenges posited by COVID-19 and even to benefit from the crisis (Guo et al. 2020). The dynamic nature of digital communication has continuously challenged human resource management in a unique way, especially in recruitment and selection (Holland and Jeske 2017). Increasingly, companies have started using social media to screen and select new applicants (Kluemper et al. 2012; Lam 2016; Henderson 2019). Research suggests that many employers consider social media sites a valuable source of information about job candidates (Peluchette and Karl 2009), which potentially allows them to draw conclusions on factors like job performance or withdrawal (Roth et al. 2016) at low costs (Clark and Roberts 2010; Brown and Vaughn 2011; Chauhan et al. 2013; Jeske and Shultz 2016). Social media profiles appear to contain more revealing information on candidates than traditional recruiting methods because users are believed to be more honest on social networks than in the résumé. Furthermore, these profiles often include information posted by others, which goes beyond the information provided and controlled by the applicants themselves (Slovensky and Ross 2012; Thomas et al. 2015). This way, the use of social media sites in recruiting decisions may prevent irresponsible employee behaviour and help the company avoid negligent hiring lawsuits, which may occur if an applicant is hired who causes harm to others and if the hiring company does not use information from background checks (Woska 2007; Elzweig and Peeples 2009; Davison et al. 2012; Bentley 2013; Kluemper 2013; Lam 2016).

While the benefits of using social media screening appear enticing for the employer, various researchers point to the risk of social media screening causing or at least facilitating unethical hiring practices (Black and Johnson 2012). Scholars have identified and discussed a number of legal and ethical issues related to the practice of social media screening, which may influence hiring decisions (Brown and Vaughn 2011; Kluemper 2013; Jeske and Shultz 2016). Given the large number of active users on platforms like Facebook or LinkedIn, the practice of screening applicants online has the potential of affecting both applicants and employers to a great extent and to have significant impact on HR decisions, such as recruiting, training, promotion, and termination (Brown and Vaughn 2011). If recruiters find information about applicants on the social web, which they think is unacceptable, applicants may be refused (Clark and Roberts 2010). This may apply to provocative or inappropriate photographs, display of poor communication skills, pictures showing alcohol or illegal drug use, false information about the applicants’ qualifications, or negative comments on previous employers or colleagues (Brown and Vaughn 2011). Even traits like self-absorption and having strong opinions displayed on social networks have been found to be viewed negatively (Tews et al. 2020), while factors as simple as language, spelling, and grammar can equally influence an employer’s impression on the applicant’s intelligence, competence, and employability (Scott et al. 2014; Becton et al. 2019). Furthermore, it is possible for other people to post embarrassing content about a person on social networks–for example, when leaving comments on other people’s profiles, or “tagging” friends in photos or videos (Jones and Behling 2010). The benefits of obtaining information at low cost and avoiding negligent hiring lawsuits thus stand in contrast to the risk of being held liable for violating applicants’ privacy and engaging in a non-validated recruitment and hiring practice, provoking adverse impact and discrimination (Jeske and Shultz 2016).

As the landscape in which companies operate is characterized by increased digitalization and growing opportunities for surveillance (Flyverbom et al. 2019), research should pay attention to concerns like surveillance and rising inequalities resulting from major changes in business and society, such as the COVID-19 pandemic (Bapuji et al. 2020). In a recruitment context, researchers need to address the relevant concerns regarding the use of social media in the selection process, as an increasing number of employers engage in such practices (Becton et al. 2019). Despite the above-mentioned issues, social media screening does not seem to be well understood by researchers and practitioners (Roth et al. 2016). A number of authors (Brown and Vaughn 2011; Davison et al. 2011; Black and Johnson 2012; Black et al. 2012; Kluemper et al. 2012; Becton et al. 2019) point to a research gap in this field. In particular, there seems to exist a lack of clear guidelines or best practices on the use of social media in selection and recruitment (Davison et al. 2012; Landers and Schmidt 2016). While the role of effective selection in successful business conduct has been largely researched with respect to traditional selection methods, not many studies have dealt with the role of social media in successful selection procedures so far. Therefore, little is known to what extent employers possess the skills and abilities to perform systematic, fair, and equitable social media screening, as noted by Hoek et al. (2016). Those studies reporting negative applicant reactions (Stoughton et al. 2015; Drake et al. 2016; Hurrell et al. 2017; Jeske and Shultz 2019) raise the question whether this practice is just and fair, and whether employers should engage in it.

Applicants’ reactions towards (in-)justice can have significant impact on individual and organizational outcomes such as job acceptance decision-making, job satisfaction, psychological well-being of applicants, self-esteem, recommendation intentions, loyalty, commitment, compliance, cooperativeness, organizational citizenship behaviour, the quality of the applicant pool, performance, and overall post-hire attitudes and behaviour (Gilliland 1993, 1994; Ployhart and Ryan 1997, 1998; Conlon and Ross 2012). As past research on the justice of social media screening has demonstrated, the perceived justice of this practice represents an important factor for job applicants (Cook et al. 2020). For this reason, it is the aim of this article to evaluate whether employers’ engagement in social media screening is acceptable from a fairness point of view. Thereby, the concept of procedural justice is used as a framework guiding the argumentation. Procedural justice evaluates the fairness of processes guiding allocation behaviours or judgments by which outcomes are attained (Gilliland 1994; Cropanzano and Ambrose 2002). Drawing from the findings of this discussion using the concept of procedural justice, an overview of recommendations for employers wishing to use social media screening will be presented. The article is organized as follows: In the first section, the concept of procedural justice will be introduced. After a brief methodological description, the procedural fairness of social media screening will be discussed according to Leventhal’s (1980) procedural justice rules. In this step, recommendations for practitioners will be outlined for each rule of procedural justice. The article closes with a summary of the results of the discussion, key takeaways for practitioners, and recommendations for further research on the use of social media in selection.

## Procedural Justice

Justice has been studied by scholars from many different perspectives. Of all disciplines concerned with studying justice, philosophy has made the most significant contribution to an understanding of justice as an aspect of morality (Greenberg and Bies 1992; Buchanan and Mathieu 2013). In ancient philosophy, justice was used to describe “a state of harmonious balance within each individual and with the social community” (Singer 2000, p. 189). The understanding of justice as an essential element of morality was at that time mainly influenced by the ideas of Plato and Aristotle (Greenberg and Bies 1992; Singer 2000). Thereby, Plato’s *Republic* and Aristotle’s *Nichomachean Ethics* represent early works on distributive justice (Cohen 1987). For Aristotle, justice represents the “the sum of all Virtues” (Singer 2000). In Book V of *Nichomachean Ethics*, he distinguishes distributive justice, which is the justice of just distribution between individuals, from rectificatory justice, which refers to restoring a disturbed relationship between individuals because of a wrongful act (Greenberg and Cohen 2014). Although Aristotle developed a complex concept of what he called “the good life,” which incorporated social-system justice, he provided no guidelines as to how to attain it (Stephens and Cobb 1999).

According to Kantian philosophy, justice in allocation seeks a balance between people’s entitlements and their moral duties, based on pure reason (Singer 2000). Kant took a deontological perspective, with the duty to respect the moral rights of others, regardless of the consequences in view of the overall good (Demuijnck 2009). One of the most influential justice theorists in modern philosophy is John Rawls (Greenberg and Bies 1992; Simola 2003; Kittay 2018). In his pathbreaking work “A Theory of Justice” (1971), Rawls attempts to obtain consensus about the meaning of justice by defining “Justice as Fairness” (McGowan 1990). Rawls establishes a conception of justice, based upon the theories of the social contract as postulated by Locke, Rousseau, and Kant (Konow 2003; Ege and Igersheim 2010), partly criticising the notion of Utilitarianism. In Rawls’ theory, the concepts of equality and duty, including the duty to help the needy, are central elements of justice (Konow 2003). In his theory, Rawls (1971) postulates two key principles: the principle of equality in the distribution of political and civil liberties, and the so-called difference principle, which requires inequalities in the distribution of social and economic goods to exist only to an extent to which they would make the least advantaged better off than under strict equality (Cohen 1987). In contrast to the more recent distinction of distributive and procedural justice, Rawls’ understanding encompasses both process and outcome (Singer 2000). Other scholars, such as the psychologist Lawrence Kohlberg (1973, 1981), were largely influenced by Rawls’ theory of social justice (Simola 2003).

Justice has been recognized as a critical element of ethical thought, which is particularly applicable to the ethics of business conduct (McGowan 1990). The study of business ethics is inherently prescriptive from a philosophical perspective, as it aims to determine what needs to be done to accomplish justice. In contrast, from a social science perspective, it is rather descriptive in describing people’s attitudes and behaviours with respect to justice (Greenberg and Bies 1992). In psychology and organisational theory, several positive theories of justice explain either the fairness of the final allocation outcome (which refers to outcome justice) or the fairness of the allocation procedures (which is procedural justice) (Singer 2000). Organizational justice most commonly refers to people’s subjective perception of fairness in organizations (Colquitt 2001). As a field of research, it represents endeavours aiming to explain the impact of justice on organizational functioning (Greenberg 1987, 1990b). Justice research has identified four dimensions of justice: distributive, procedural, interpersonal, and informational justice (Colquitt 2001; Loi et al. 2012; Xu et al. 2016). Thereby, interpersonal and informational justice are subgroups of the concept of interactional justice, as established by Bies and Moag (1986).

Initially, organizational justice research was interested in the perceived justice of decision *outcomes,* which is distributive justice (Gilliland 1993; Hartman et al. 1999; Colquitt et al. 2001). Research on distributive justice is especially concerned with the question of whether such outcomes are consistent with implicit norms for allocation, like equity and equality (Adams 1965; Colquitt 2001; Cropanzano and Ambrose 2002). Equity describes the ratio of inputs to outcomes for an individual being relatively equal to the input-outcome ratio for others (Adams 1965; Cohen 1987; Gilliland 1994; Alder and Gilbert 2006). This concept was introduced in Adams’ (1965) study of Equity Theory, which incorporated a ratio of what individuals receive relative to what they put in when they work for an organization. Comparing this ratio to the ratio of other individuals allows them to make judgments on the fairness of their own ratio (Cropanzano and Randall 1993). However, practice revealed that the resources managers can distribute to employees are limited, which is why employees cannot always receive all rewards they deserve (Shapiro 1993). It was realized that the way an individual is treated in the allocation *process* is just as important to the individual as the actual outcome. In fact, the favourability of a certain outcome can matter less as long as the allocation process is fair, because the individuals being subject to the process may expect that a system which functions that way may meet their needs in the future (Cropanzano and Randall 1993). Thus, if an individual is treated poorly in the selection process, this person may expect that the organization generally treats employees poorly (Gilliland 1993) and may consider it dysfunctional (Alder and Gilbert 2006). Empirical findings on positive theories of justice even suggest that people are generally more concerned with the fairness of the allocation procedure than that of the outcome (Lind and Tyler 1988; Singer 2000). Therefore, the study of the factors that influence perceptions of the fairness of the allocation process beyond the mere distribution of more resources to employees may broaden managers’ possibilities of distributing rewards (Shapiro 1993). In consequence, research interest shifted from the study of the fairness of results towards the study of the fairness of the process by which such results are attained (Hartman et al. 1999), which is procedural justice (Gilliland 1994; Cropanzano and Ambrose 2002). A procedure is understood as a sequence of steps that guide allocation behaviours or judgments. Individuals who participate in such procedures generally form an opinion about their fairness (Cropanzano and Ambrose 2002). Unlike distributive justice, which neglects the social system that generates a certain distribution, procedural justice is related to the evaluation of systems or institutional characteristics (Leventhal 1980; Folger and Konovsky 1989), which is one reason why it was chosen as a framework to evaluate the fairness of social media screening in the selection process.

Another reason why procedural justice was chosen as a framework for this article is the existence of general consent in the literature that procedural justice affects employee behaviours and attitudes (Lind and Tyler 1988; Greenberg 1990b). Procedurally fair processes have been found to be more profitable than unjust processes because they serve an economic benefit (Cropanzano and Ambrose 2002). Thus, a great amount of research has been conducted on the positive influence of perceived procedural justice on a breadth of positive employee attitudes and behaviours at work (Brebels et al. 2010), as well as organizational outcomes such as satisfaction, commitment, trust, performance (Nowakowski and Conlon 2005), withdrawal, negative reactions, evaluation of authority, and organizational citizenship behaviour (Colquitt et al. 2001). Several studies have even shown that the negative effects of perceived unjust outcomes may be mitigated by increased procedural fairness (McFarlin and Sweeney 1992; Sheppard et al. 1992; Vermunt and Steensma 2001; Alder and Gilbert 2006). In a hiring context, individuals rejected for a job may be more likely to accept the decision if they consider the company’s selection procedures fair, even though they perceived a breach of distributive fairness by thinking of themselves as qualified for the position. In turn, even individuals who were given a job may develop a negative attitude towards the hiring organization if they perceive the selection process as unfair (Alder and Gilbert 2006). It should therefore be in the interest of any organization to consider the perceived fairness of its recruitment methods very closely (Cook et al. 2020).

The study of procedural justice was introduced by Thibaut and Walker (1975), who examined disputant reactions to legal procedures through third-party intervention. While this work, along with other contributions that followed, concentrated on procedural justice in a legal context (Colquitt et al. 2001), it was Leventhal (1980), who introduced the concept of procedural justice to non-legal contexts such as organization research. To set standards for the evaluation of procedural justice, Leventhal (1980) proposed six rules of fair procedure: consistency, bias suppression, accuracy, correctability, representativeness, and ethicality. As noted by Cropanzano and Greenberg (1997), as well as Cropanzano and Ambrose (2002), Leventhal’s rules may have undergone modifications to be adapted in different contexts over time, but still have proven to be useful. For this reason, this paper aims to discuss procedural justice with respect to social media screening according to Leventhal’s justice rules.

## Methodology

The first aim of this article is to discuss whether social media screening violates the principles of procedural justice. Second, it seeks to articulate recommendations for practitioners wishing to perform social media screening in a fair manner. To achieve this, the article presents a review of the main benefits and criticism of social media screening identified in the literature and discusses them using Leventhal’s (1980) rules of procedural justice. To assess the state of research on social media screening, the procedure recommended by Webster and Watson (2002) was applied: First, publications were revealed from electronic databases using the search terms “social media screening,” “social network screening,” “social media assessment,” “social network assessment,” as well as “social media in selection.” Secondly, the literature identified within electronic databases was screened for further articles cited in these works. Thirdly, those articles citing the works identified in the first step were reviewed. All articles were then screened for benefits, issues, and recommendations pertaining to the use of social media screening. The results from this step were then classified according to Leventhal’s (1980) rules: consistency, bias suppression, accuracy, representativeness, correctability, and ethicality. According to Cropanzano and Ambrose (2002), the rule of representativeness is often equated with the principle of “voice”, which considers the importance of two-way communication in procedural justice, as established by Thibaut and Walker (1975). Other authors, such as Zapata-Phelan et al. (2009) treat voice as a separate rule. In contrast to mere representation, voice allows for providing direct input, thereby giving individuals the opportunity to exert direct control over the decision-making process (Zapata-Phelan et al. 2009). Therefore, voice is included as a separate rule in this article.

Several limitations have to be considered regarding the scope of this review. While social media is used in recruitment, selection, and disciplinary situations (Lam 2016), it is important to distinguish between the processes of recruitment and selection: Recruitment is concerned with activities aimed at attracting an acceptable group of candidates to a certain position, whereas selection is concerned with choosing the most suitable person for a position from a pool of candidates through the use of assessment tools (Aguado et al. 2016). As Lam (2016) notes, the ethical issues related to using social media in the selection process are more complex and serious than for the recruitment process. Tensions may also arise from the implications of monitoring and disciplining employees or employees’ private use of social media during worktime after they have been hired (McDonald and Thompson 2016). These matters will, however, also be excluded from this paper. While organizations certainly face ethical issues when it comes to monitoring current employees’ social media activities (Lam 2016), the ethical and legal issues emerging in that context differ from those in selection and should be dealt with separately. The following discussion will therefore concentrate solely on the use of social media in selection and will exclude all matters pertaining to recruitment, such as job advertising on social media, or post-hire situations involving social media.

Another limitation concerns the search scope in the online screening process. While employers have increasingly started using social media for HR purposes (Lam 2016; Henderson 2019), the search for applicant information may also be extended to a general internet search, for example, by using search engines (Roth et al. 2016). While potential overlaps may exist between a more general internet search and the exclusive use of social media, authors like Kluemper et al. (2015) as well as Roth et al. (2016) make a clear distinction between a general internet search that makes use of a variety of sources, and the use of social media websites only. Kluemper et al. (2015) argue that social media display specific issues relating to impression management, discrimination, privacy, and applicant reactions that may not apply to general internet searches because they enable limitations in the accessibility to the site and to individual user profiles. Following this argumentation, this article concentrates exclusively on the ethicality of consulting social media sites and disregards the implications of a broader, more general internet search that would include a wider variety of online sources.

## The Procedural Justice of Social Media Screening

For the employer, the practice of social media screening has a number of obvious advantages. Literature suggests the use of social media enables the employer to efficiently and conveniently review readily available information at low costs, which allows for a wide range of organizations to take advantage of this practice, including small businesses (Brown and Vaughn 2011; Chauhan et al. 2013; Jeske and Shultz 2019). Another major reason for using social media in the selection process is that social media profiles potentially contain more revealing information on a candidate than traditional application documents: Firstly, because users are believed to be more honest on social networks than in the résumé, and secondly, because social media websites often include information posted by others, which goes beyond the information provided and controlled by the applicant him- or herself (Slovensky and Ross 2012; Thomas et al. 2015). Through this allegedly more authentic representation on social media, the screening process may then present HR professionals with the opportunity of drawing conclusions about the applicant’s character and personality, which cannot easily be done by traditional means (Kluemper and Rosen 2009; Brown and Vaughn 2011; Chauhan et al. 2013). According to Nikolaou (2014), social media are typically used to find additional information about candidates after receiving their application documents or after the first interview. This additional information may assist recruiters in verifying application documents such as the résumé (Brown and Vaughn 2011). In particular, Kluemper and Rosen (2009) note that information on social media may be effective in drawing conclusions on applicants’ so-called “big-five” personality traits: agreeableness, conscientiousness, extraversion, neuroticism, and openness to experience. Such conclusions can be based on the types of interest groups applicants have joined, the things they “like”, status messages, comments, or tagged photos. These activities are assumed to reflect regular behaviour, as opposed to rehearsed behaviour, which would perhaps occur in a job interview setting. A study by Rosen et al. (2018) on the use of Twitter profiles to assess candidates’ personality and suitability found out that Twitter profiles can accurately predict personality, even when used as a sole predictor. This way, employers may find individuals who possess the characteristics the company deems desirable and who identify with the company’s goals and objectives (Rosen et al. 2018). Hence, it allows companies to assess applicants’ potential fit with the organization (Becton et al. 2019), thereby reducing uncertainty about candidates and predicting future communication outcomes (Evuleocha and Ugbah 2018). If applicants are considered unsuitable, they may be screened out based on the information from social networks (McDonald and Thompson 2016). Therefore, another frequent argument for the use of social media assessment is that the company bears a certain responsibility towards its stakeholders when making hiring decisions to reduce the risk of irresponsible or even criminal employee behaviour (Kluemper 2013). For example, this may be true if an applicant to a day-care centre “likes” content oriented toward paedophilia (Slovensky and Ross 2012), or if an applicant to a position as a transportation worker is portrayed online with many posts involving parties and alcohol (Davison et al. 2016). Past behaviour can be a good predictor of future behaviour when it comes to the discovery of illegal or harmful activities that may later cause damage to co-workers or customers. Even if the applicant did not engage in criminal behaviour, the company may discover indicators of disloyalty, for instance in the form of blackmailing past employers or spreading trade secrets (Slovensky and Ross 2012). If an organization knowingly hires an applicant who causes harm to others and if that organization does not use information from background checks, it can be sued for negligent hiring (Woska 2007; Elzweig and Peeples 2009; Davison et al. 2012; Bentley 2013; Kluemper 2013; Lam 2016), especially when the organization is involved in public safety, such as trucking, child care, or public sector activities (Slovensky and Ross 2012). Therefore, the information obtained from social media assessment is often used by companies in order to avoid negligent hiring lawsuits (Chauhan et al. 2013).

Despite its great potential of improving hiring decisions, the use of social media in selection raises several legal and ethical concerns (Thomas et al. 2015). In this context, opponents of social media screening are particularly concerned with potential privacy and discrimination issues (Black and Johnson 2012; Black et al. 2015; Thomas et al. 2015; Lam 2016). Research suggests that social media screening causes strong moral reactions in job candidates due to potential invasions of privacy (Black et al. 2015). Often, social networking websites contain personal information about applicants, which is not allowed to be asked during a job interview such as race, marital status, or sexual orientation. If the employer accesses this sensitive information in an online search on social networks, this may lead to a negative employment decision on that candidate (Jones and Behling 2010). It is therefore not surprising that authors like Clark and Roberts (2010) deem social media screening a socially irresponsible practice. Another issue frequently criticized by researchers and commentators is the accuracy of information from social media, as such information on candidates may be untrue, incomplete, or taken out of context (Slovensky and Ross 2012; Lam 2016).

A reward distribution process like the hiring process is often complex in nature, and involves the selection of decision makers, the structuring of the decision process, and other organizational steps (Leventhal 1980). This poses a challenge for managers pursuing just and fair decision processes, especially in a social media screening setting, which lacks clear guidelines and policy recommendations for hiring managers (Davison et al. 2012; Landers and Schmidt 2016). In their study revealing negative reactions from job applicants/employees towards social media screening, which result from perceived justice violations, Hurrell et al. (2017) stress a need for policy advice as to how employers should use social media screening. As potential negative feelings arising from the process of social media screening may be improved by paying attention to the procedural justice of this practice (Hurrell et al. 2017), the rules of Leventhal (1980) are hence applied to formulate recommendations for a fairer performance of social media screening. Thereby, potential drawbacks serve as starting points for improvements. The following sections will discuss the procedural justice of social media screening according to each of Leventhal’s (1980) rules.

### Consistency

The consistency rule requires processes to be applied consistently across persons and across time (Leventhal 1980; Colquitt 2001; Alder and Gilbert 2006). Applied across people, similar procedures must be performed for all potential recipients of a reward without giving preference to anyone. This rule is therefore related to the concept of equality of opportunity (Leventhal 1980), requiring the hiring manager to treat similarly situated people in a similar manner with regard to process and outcome (Alder and Gilbert 2006). Also over time, the way a process is handled should be kept stable, at least over the short term. If a process is applied differently to different people or changes are made too frequently or too easily, this may lead individuals to conclude that procedural fairness is violated (Leventhal 1980). While acknowledging that the people who possess the necessary skills for the job may be situated differently to those who lack those skills, Alder and Gilbert (2006) highlight that hiring managers should give the possibility to obtain a certain job consistently to those individuals most qualified for the job. Thereby, they should proceed with care and rigour, paying attention to diligent procedures.

When the consistency rule is applied across people, Bentley (2013) discusses the possibility of social media screening to be discriminatory on the basis of protected characteristics. This may be the case when metadata reveals that a hiring manager spent more time on the profile of a white applicant, and significantly less time viewing the social media profile of an African-American applicant, and then decides to hire the white applicant. In this example, the hiring manager screens applicants differently based on race, which would represent a case of disparate treatment. As screening only certain applicants can be understood as a form of discrimination, it is recommended to perform the screening for all applicants (Bentley 2013; Davison et al. 2016). Since employers’ screening of social media in selection often occurs ad hoc and in an arbitrary manner (Schroeder et al. 2020; Wade et al. 2020), organizations should make efforts to standardize the process (Davison et al. 2016; Landers and Schmidt 2016; Tews et al. 2020; Wade et al. 2020) in order to give all participants the chance to obtain positive results. Overall, a considerable number of authors point to the necessity of articulating a clear, standardized policy for carrying out social media screening (Elzweig and Peeples 2009; Davison et al. 2012, 2016; Lam 2016; Landers and Schmidt 2016; Wade et al. 2020) and disseminate it to the employees (Elzweig and Peeples 2009). This policy should specify the conditions under which social media content should be collected, differentiate job-relevant from irrelevant and discriminatory information, and establish a standardized rating system to account for more objectivity (Tews et al. 2020; Wade et al. 2020). Despite the emphasis placed by various authors on the need to structure the process of gathering applicant information via social networks, the study of Schroeder et al. (2020) found that adding structure to the process did not lead to the expected improvements in psychometric properties demonstrated in social media screening. In reaction to these findings, Schroeder et al. (2020) presume that structuring methods other than those used in their study may be more effective. As a result, future research on the effectiveness of different methods of stucturing the process of social media screening may provide further insights for practitioners. Table 1 provides an overview of the key recommendations for enhancing consistency in social media screening.

**Table 1:** Recommendations to enhance consistency in social media screening

Recommendations	Selected Literature
• Apply social media screening consistently to all applicants	Leventhal (1980); Colquitt (2001); Alder and Gilbert (2006); Bentley (2013); Davison et al. (2016)
• Keep the process stable over time	Leventhal (1980); Colquitt (2001); Alder and Gilbert (2006)
• Standardize the process of social media screening and establish a binding policy for social media screening	Elzweig and Peeples (2009); Davison et al. (2012, 2016); Lam (2016); Landers and Schmidt (2016); Tews et al. (2020); Wade et al. (2020)

### Bias Suppression

Individuals can base their judgments of the fairness of an allocative process upon the bias suppression rule, which requires abstention from personal self-interest and reliance on narrow preconceptions or doctrinaire views (Leventhal 1980; Alder and Gilbert 2006). Bias in the selection process may take various forms: A hiring manager may give jobs to friends and relatives or refuse to hire qualified individuals based on demographic information or personal prejudices. In such cases, fairness was found to be lowest (Singer 1993; Alder and Gilbert 2006). The bias suppression rule of procedural justice thus requires hiring managers to be neutral (Colquitt 2001).

Social media screening of applicants may induce bias (Wade et al. 2020) and discrimination (Manant et al. 2019) for a variety of reasons. One source of discrimination against applicants is the demographic information published on a social media profile. Given the wide range of personal information available on social media, such as race, gender, age, or disability status, the use of this non-professional information in making employment decisions may lead to unfair discrimination (Kluemper and Rosen 2009; Nikolaou 2014). Often, this kind of information contains answers to questions that are not allowed to be asked during job interviews (Zhang et al. 2020). Thus, making employment decisions based on such restricted information can lead to adverse hiring decisions (Jones and Behling 2010) and is often prohibited by law (Black and Johnson 2012; Bentley 2013; Roth et al. 2016). For example, the social media site may contain information revealing the candidate to be part of a protected class or category. If an employer regularly checks potential job candidates’ profiles, and the organization does not have enough workers who are members of protected classes, the organization may find itself confronted with a discrimination lawsuit (Elzweig and Peeples 2009). While it is often difficult to raise such claims, as only few cases of discrimination are brought to court (McDonald and Thompson 2016), perceived procedural fairness is nevertheless decreased if applicants feel that the hiring decision was biased (Leventhal 1980).

Alder and Gilbert (2006) point out the importance of being aware of the difference between intentional and unintentional systematic bias. As opposed to more obvious forms of discrimination based on race or age, the unintentional bias may be subjective and subconscious (Bertrand and Duflo 2017). Hiring managers may not only reject a candidate on the grounds of demographic information, but also when they find a piece of personal information that corresponds to their stereotypes (Jones and Behling 2010). Hiring managers’ biases may thus (wrongly) frame their picture of a candidate, even without malicious intent (Davis 2007; Elzweig and Peeples 2009). Especially non-professional sites like Facebook offer their users prompts to enter a wealth of personal information (for example, activities, interests, relationship status, favourite music, and a profile picture), as well as the freedom to post additional information or media (Karl et al. 2010; Chauhan et al. 2013). Such job-irrelevant content may induce biases related to the values, attitudes and beliefs of an applicant. For example, if recruiters find information on applicants’ political views (Wade et al. 2020) or, if a candidate likes a certain sports team the employer hates, the stereotype of the employer may lead to a negative hiring decision (Jones and Behling 2010). The research of Wade et al. (2020) demonstrates that employers tend to rate applicants higher in a social media screening situation when they feel that those applicants are similar to them. While these results mainly pertain to political views, the described phenomenon may also occur if applicant and recruiter like the same sports team or went to the same high school (Alder and Gilbert 2006). Likewise, physical attractiveness based on photos posted on social media may lead to unfair biases (Alder and Gilbert 2006; Jones and Behling 2010; Black and Johnson 2012). Thereby, research suggests that the effect of physical attractiveness is stronger for women than for men, as attractiveness is related to femininity rather than to masculinity. Therefore, women are held to higher standards for physical attractiveness than men (Black and Johnson 2012). Applicant sex as such may also influence the way information from social networks is used by recruiters during selection (Alder and Gilbert 2006; Becton et al. 2019). Overall, due to personal biases and stereotypes, the same piece of profile information may provoke different perceptions of a candidate among different raters, thereby raising or lowering an applicant’s chances of being hired (Davis 2007). In addition to such individual stereotypes, Becton et al. (2019) contrast the influence of unprofessional social media content on employers’ potential negativity biases and the effects of professional or positive social media content on employers’ perceptions of candidates. They point out that negative information generally has a stronger influence on people’s perceptions than comparably extreme positive information. Thus, recruiters presented with unprofessional social media content may expect that applicant to exhibit more undesirable behaviour on the workplace than positive information would create a favourable impression of a candidate.

The absence of social media information on an applicant may also be a source of discrimination, because not all individuals are similarly or frequently active online (Thomas et al. 2015; Jeske and Shultz 2016). Applicants who maintain a social media profile may be perceived more positively than those without a social media profile (Black and Johnson 2012; Slovensky and Ross 2012; Alexander et al. 2019). Research suggests that missing information may lead to increased uncertainty about the attributes of applicants, which then results in the devaluation of a candidate’s skills as compared to those who provide such information (Roth et al. 2016). Several studies have shown differences in the adoption and usage of social networks across gender, age, as well as occupational groups. There may exist an underrepresentation of minorities on social media, and women and elderly applicants may also be perceived less favourably than men and younger applicants (Black and Johnson 2012; Alexander et al. 2019). Older individuals may be less proficient in the use of technology (Chauhan et al. 2013) and are therefore less likely to be active on social media. This could lead to an adverse impact if an employer is not able to find any information on an older candidate (Davison et al. 2012; Kluemper et al. 2015). In addition to older individuals tending to be less active on social media, certain socioeconomically disadvantaged racial or ethnic groups may have no or limited access to the internet (Davison et al. 2012; Kluemper et al. 2015). Furthermore, there may also be potential gender differences in the use of social media (Kluemper et al. 2015). For instance, research by Zide et al. (2014) revealed that men are more active in sharing personal and professional information and making recommendations on social networks than women. Authors like Nikolaou (2014) and Alexander et al. (2019) confirm these findings by noting that men are more active on the professional social network LinkedIn than women. Furthermore, the study by Nikolaou (2014) shows that certain occupational groups are more likely to perform well on presenting themselves on social media. For instance, sales and marketing professionals were perceived to be better at presenting themselves online than other professional groups because it is part of their training. However, adoption and usage of social networks not only differ based on a lack of access or proficiency, but are also a matter of choice. Users may consciously decide to discontinue social media temporarily or permanently. One reason is the phenomenon of social network fatigue, which refers to negative emotional reactions, like tiredness, boredom, burnout, indifference, or decreased interest resulting from social media activities (Zhang et al. 2016). People feel increasingly pressured to respond to an overwhelming volume of social demands on social networks, which leads to physical and psychological strain (Lee et al. 2016). Since people change their behaviour in order to avoid such stress, social network fatigue has been found to be one reason why users discontinue using social media (Maier et al. 2015). Studies like the ones conducted by Xiao et al. (2020), and Wang et al. (2021) demonstrate that this energy-consuming social overload along with invasion of privacy are key enablers that incite users to discontinue social media use. While this phenomenon has received little attention in research on social media so far (Wang et al. 2021), its implications should not be disregarded in this context. As Xiao et al. (2020) call on providers of social media sites to be considerate of users’ processing capability and to give users complete rights over the information they post to protect their privacy, it seems likewise reasonable to expect hiring companies to respect applicants’ needs to protect themselves and make efforts to avoid discrimination based on missing social media information.

In order to prevent discrimination and unfair biases, the timing of the screening, as well as the choice of individuals conducting the screening play a key role. See Table 2 for a summary of the recommendations from the literature. First of all, it is recommended to perform the social media screening late in the process when obvious protected class memberships are already known (Davison et al. 2016). Since the pieces of information obtained from social media may be perceived differently across raters (Davis 2007), it is necessary to use multiple raters (Davison et al. 2012, 2016; Kluemper et al. 2012). However, the task of online background checks should only be done by a limited pool of authorized individuals (Thomas et al. 2015; Lam 2016). According to Davison et al. (2016), it should be the task of the HR department to do social media checks, as HR professionals are more familiar with issues of validity, adverse impact, and disparate treatment than practicing managers, who may also be too tempted to examine non-job-related factors. By separating the individuals conducting the social media assessment from those conducting interviews, information can be prevented from being shared between separate assessors (Davison et al. 2016). Also, Lam (2016) recommends separating the roles of the person collecting the data from the person making the employment decision so that information cannot be modified prior to entering the decision process. Furthermore, the social media assessors should receive training on how to screen and gather social media information (Elzweig and Peeples 2009; Thomas et al. 2015; Wade et al. 2020). By training the social media assessors in what to search for (for example, to concentrate on job-related information), the organization can potentially safeguard itself against bias (Davison et al. 2016).

**Table 2:** Recommendations to reduce bias and discrimination in social media screening

Recommendations	Selected Literature
• Perform the social media screening late in the process	Davison et al. (2016)
• Use multiple raters	Davison et al. (2012, 2016); Kluemper et al. (2012)
• Limit the pool of individuals authorized to perform online background checks	Thomas et al. (2015); Lam (2016)
• Have the HR department do the social media screening	Davison et al. (2016)
• Provide training for social media assessors on how to gather job-related information from social media	Elzweig and Peeples (2009); Thomas et al. (2015); Davison et al. (2016); Wade et al. (2020)
• Separate the individuals conducting the social media screening from those conducting interviews	Davison et al. (2016)
• Separate the individuals conducting the social media screening from those making the employment decision	Lam (2016)

### Accuracy

The accuracy rule of procedural fairness requires an allocative decision to be based on high-quality information and informed opinion. Thus, the information and opinion upon which a decision is based must be obtained with great care to reduce error to a minimum (Leventhal 1980).

The practice of social media screening in selection raises data accuracy concerns in several ways: In their study, Schroeder et al. (2020) demonstrate that the assessments of applicants based on information from social media screening were generally inconsistent with the information provided by the applicants. Regarding the quality and reliability of social media information, Davis (2007) has identified the problem of inaccurate or false information about candidates, especially beyond their own control, as being one of the most serious issues in social media screening. It may result in poor employment decisions, creating an inaccurate or unfair impression of candidates (Davis 2007). This constitutes a risk for the organization when the “wrong” person is hired, and is unfair to individuals who were turned down based on false information (Lam 2016). There are various possible sources of false information: One is false information on a candidate provided by others. Part of the information about a person available on social media is submitted by other people, such as comments on the “wall,” or “tagged” photos or videos, whereby a person’s name is attached to a photo or video, which can then be found by searching that person’s name (Jones and Behling 2010). This may be done even without that person’s consent (Chauhan et al. 2013). Some of this information can be deleted by the user, but control over content submitted by others is limited (Kluemper and Rosen 2009). As a consequence, users can become victims of identity theft (Connerley et al. 2001; Davison et al. 2012) or libel, whereby false and defamatory information is posted on their websites or profiles (Davison et al. 2012). Entire fake profiles of candidates can be created by other people without the knowledge of the victim (Davis 2007). When taken out of context, even correct information can be misleading (Kaupins and Park 2011), for instance, when “tagging” friends in photos (Chauhan et al. 2013).

While some aspects of social media profiles are difficult to fake, such as the basic demographic information (Black and Johnson 2012), users themselves may also become a source of false or inaccurate information. Since making a positive impression with one’s social media appearance may be helpful in succeeding in the application process (Hoek et al. 2016), it is not surprising that some applicants clean up their social media profiles (Davison et al. 2016) and try to make them look more favourable if they know that a potential employer is looking (Kluemper and Rosen 2009; Black and Johnson 2012; Chauhan et al. 2013; Jeske and Shultz 2016). In this context, Jeske and Shultz (2016) refer to the concept of role distance (Goffman 1961, 2005), wherein individuals enact different roles, depending on the expected audience. By assuming such a role, they can distance themselves purposefully from certain groups to appeal to an intended audience or create affiliation with certain desired social hierarchies and groups. Depending on the audience, users may remove negative contents or fake the information they post on a social media webpage. For example, they may “fake good” if they expect their parents or employers to view the page, or “fake bad” to impress friends (Davison et al. 2011, 2012). Some users even create a public page with their real name and a separate “pseudo site” for their friends, which contains content specifically targeted at them (Clark and Roberts 2010). As a result of this kind of impression management, chances are that potential employers are unable to obtain the expected information (Cook et al. 2020) and may misinterpret fit (Hoek et al. 2016).

Due to its particular relevance for the process of gathering information about potential receivers of reward, more than to any other procedural component, the accuracy rule is key for a fair selection process. Following Leventhal’s (1980) accuracy rule, employment tests that do not reliably predict future performance on the job would constitute a violation of procedural fairness. In the attempt to identify the candidate who will best perform on the job, perceived job relevance, even more than actual job relevance, serves as another indicator for perceived selection fairness (Gilliland 1994; Alder and Gilbert 2006). Research has shown that applicants prefer job-related procedures (Stoughton et al. 2015): While biographical and personality inventories are effective methods of predicting job performance (Hunter and Hunter 1984; Alder and Gilbert 2006), they are often considered unfair because they rely on questions about applicants’ personal lives and mental health, which are questionable in their relation to the job (Smither et al. 1993; Alder and Gilbert 2006). Thus, applicants perceive work sample tests and interviews more favourably than personality tests and biodata/weighted application forms because the link to job relevance is more obvious (Hausknecht et al. 2004; Slovensky and Ross 2012). Since Slovensky and Ross (2012) observe an apparent similarity between social media screening and biodata, as both measure background, history, and outside interests that are not obviously job-related, the authors consider social media screening to be perceived to perform rather low in procedural justice. They conclude that social media screening may be viewed as unfair, even for applicants who are offered the job.

Since objective data about the accuracy of the selection process is rarely available to applicants, they must turn to other indicators of accuracy, one of which is transparency (Schuler 1993; Alder and Gilbert 2006). In this context, a selection tool is transparent when its job relevance is apparent to the applicant. Conversely, transparency is low when job relevance is less clear (Alder and Gilbert 2006). Concerns have also been raised about the validity of information with little job relevance, which is produced as part of social media assessment (Roth et al. 2016). For example, pictures and other pieces of information on social media can be misleading: Someone pictured obviously intoxicated at a party may still be a good employee, irrespective of what he or she does beyond working hours (Chauhan et al. 2013). While that person may not necessarily be an alcoholic, not everyone holding a hunting rifle in a picture is a mass murderer (Elzweig and Peeples 2009). Therefore, the question remains if the information obtained from a social media profile really paints an accurate picture of applicants in their professional lives.

In the literature, there exist several proposals of enhancing information accuracy, which are summarized in Table 3. The first recommendation is to validate the results of the social media assessment upon reliance on traditional selection methods (Landers and Schmidt 2016), as traditional methods generally enable more cognitive and rational assessments of applicant information and base suitability on job-relevant information (Wade et al. 2020). Thereby, one first step is to conduct a job analysis to ensure the job-relatedness of the data collected on social media (Davison et al. 2016; Landers and Schmidt 2016). Based on this job analysis, the factors that are clearly relevant for examination in social media screening (Tews et al. 2020) should be determined beforehand. For example, in personality tests, the “big-five” measures of personality are measured for selection purposes. While examining for extraversion may be useful in hiring a sales representative, it may be less so for university staff. Thus, when only those factors are used that are effective predictors of job-relevant performance, transparency can also be enhanced (Alder and Gilbert 2006). Questions concerning the scope of the social media search should also be clarified in advance: For instance, it should be determined whether or not to limit the search to the applicants’ sites or to extend it to their lists of friends (Slovensky and Ross 2012). To increase job-relatedness of information obtained through social media, several authors recommend limiting the scope of social media sites used for screening to professionally-oriented networks, such as LinkedIn, because these types of networks are job-related and less focussed on personal information (Thomas et al. 2015; Hurrell et al. 2017; Cook et al. 2020).

Another major factor in achieving improved procedural justice in the process of social media screening is to ensure transparency (Hurrell et al. 2017). Personality tests, biodata, and cognitive tests, which are comparable to social media screening, are effective in predicting performance and aid in hiring decisions. Therefore, Alder and Gilbert (2006) recommend that they should not simply be excluded from use because of low transparency, as there are ways of improving the transparency of such selection tools. To achieve this, record keeping has been identified as a key method of monitoring the people who control the allocative process, mostly for inspection by concerned parties (Leventhal 1980; Lam 2016) and to provide support in the case of a litigation (Landers and Schmidt 2016). Such records should clearly document which websites should be viewed, which behaviours should be predicted, and how the ratings of the assessors are made (Davison et al. 2016). If a hiring tool is less transparent, it is also recommended to explain it (Alder and Gilbert 2006). Also, the procedure, measures, and criteria underlying the decision should be communicated to the applicant accordingly (Alder and Gilbert 2006; Conlon and Ross 2012; Aguado et al. 2016). Thereby, quality and timing of an explanation play a role in how credible and sincere an explanation is perceived (Conlon and Ross 2012). Generally speaking, the earlier information is received, the greater the impact on the justice perception (Van den Bos et al. 1997). In case of an unfavourable outcome, an explanation received in advance may allow the recipient to interpret the outcome in the context of the explanation (Weaver and Conlon 2003). It is thus advisable to provide explanations for the rationale behind the decision-making process as early as possible, ideally before the decision is made.

**Table 3:** Recommendations to enhance accuracy in social media screening

Recommendations	Selected Literature
• Validate the results of the social media assessment against traditional selection methods	Landers and Schmidt (2016)
• Conduct a job analysis	Davison et al. (2016); Landers and Schmidt (2016)
• Clearly determine the job-related factors that should be subject to examination	Alder and Gilbert (2006); Tews et al. (2020)
• Clearly determine the search scope of the social media screening	Slovensky and Ross (2012)
• Limit the scope of social media sites used for screening to professionally-oriented networks	Thomas et al. (2015); Hurrell et al. (2017); Cook et al. (2020)
• Keep records and document the social media screening process at all stages	Leventhal (1980); Davison et al. (2016); Lam (2016); Landers and Schmidt (2016)
• Explain the procedure to the applicant	Alder and Gilbert (2006); Conlon and Ross (2012); Aguado et al. (2016)
• Provide explanations of the procedure before the final decision is made	Conlon and Ross (2012)

### Representativeness

According to Leventhal’s (1980) rule of representativeness, a fair allocative process reflects the basic concerns, values, and outlook of relevant subgroups of the people affected by the process. The importance of a subgroup may depend on factors like its size or prestige. The application of this rule in practice may vary greatly, yet it is generally advisable to include representatives of relevant subgroups in decision-making bodies, like committees. When individuals feel that their own interests are represented in the decision making, and their own power is involved, the process is likely to be perceived as fairer (Leventhal 1980). Applicants are often not aware that they are being watched, which is why they are also unaware of other users being watched who share their concerns. As a result, applicants who are subject to social media screening generally lack a collective representation of their worries and interests such as discrimination issues (Jeske and Shultz 2016). As it is recommended to use multiple raters in the process of social media assessment (Davison et al. 2012, 2016), representatives of relevant interest groups should be included in the pool of raters, such as an equality officer or a representative of people with disabilities. Table 4 summarizes the key recommendation for enhanced representation of interests in social media screening.

**Table 4:** Recommendations to enhance representation of interests in social media screening

Recommendations	Selected Literature
• Include representatives of relevant interest groups in the pool of raters	Leventhal (1980)

### Voice

Thibaut and Walker (1975) found that just procedures allow individuals some control over the decision-making process and over some of the eventual outcome through voice input and decision influence (Zapata-Phelan et al. 2009). Beginning with Folger (1977), justice research has started to acknowledge the role of voice in procedural justice by giving individuals the opportunity to express their opinion regarding a decision, or through effectively influencing decisions (Novelli et al. 1995). Voice has received significant research attention in several HR contexts (Alder and Gilbert 2006). Shapiro (1993) has examined the effect of procedural justice on third-party intervention in dispute resolution. She concludes that a perception of procedural justice occurs when individuals feel that they have indirect outcome control, or the opportunity of influencing a specific outcome by expressing their views (“voice”) (Shapiro 1993). By having voice in the decision making and thereby expressing one’s own opinion, the sheer possibility of being involved in the decision making creates a feeling of participation. Voice is therefore seen as a form of participative decision making (Folger 1977). Notwithstanding, voice provides less control over the decision process than actual choice. This is why it was later defined as an indirect form of participation (Greenberg and Folger 1983). Thibaut and Walker (1975) identified two types of control as evidence of how much influence disputants in a dispute resolution scenario had: process control and decision control. Thereby, process control represents control over the presentation of disputants’ arguments. The possibility of influencing what information gets heard or expressed in a decision process is referred to as process control, while outcome control is the control over the actual outcome of a decision process (Shapiro 1993). In the literature, the effect of process control is often referred to as the “voice” effect (Folger 1977; Lind and Tyler 1988; Colquitt et al. 2001).

In most social media screening contexts, applicants are unaware that the employer uses social media screening, to what extent information is gathered from social networks, and how strongly it influences hiring decisions (Jeske and Shultz 2016; McDonald and Thompson 2016). Thus, many rejected applicants are never told that an adverse decision was made on the basis of social media screening (Clark and Roberts 2010), and discrimination may occur without the applicant’s knowledge (Manant et al. 2019). Therefore, the screening of online sources is considered as an “extractive” rather than an “interactive” search for information, as it does not involve two-way communication with the applicant (Berkelaar 2014, 2017; Berkelaar and Buzzanell 2014). Clark and Roberts (2010) therefore describe employers engaging in social media screening as “undetectable voyeurs to very personal information” (p. 518). According to Slovensky and Ross (2012), applicants may perceive this kind of process as unfair due to low informational justice, as they often become aware that their social media information has been consulted by an employer only when the decision is already made. Social media screening thus seldom leaves room for two-way communication, but is frequently performed in secret and without a chance for the applicant to comment on it directly. However, as a reaction to an adverse decision, applicants can turn to social media to express voice on the perceived fairness of an organization’s selection process in public (Conlon and Ross 2012; Stoughton 2016), which has the potential of causing substantial damage to the organization’s reputation (Stoughton 2016). To avoid this scenario, organizations should provide the opportunity for two-way communication already during the selection process (Hurrell et al. 2017) and thereby allow individuals to express their voice (Shapiro 1993; Conlon and Ross 2012), especially when they receive unfavourable outcomes (Conlon and Ross 2012; Davison et al. 2016). Allowing individuals to express voice enhances their perception of procedural justice, even if they cannot directly influence the outcome by doing so (Lind and Tyler 1988; Conlon and Ross 2012). As a result, they should be more accepting of the outcome (Conlon and Ross 2012), and a perceived violation of distributive justice may be offset by increased procedural justice. Since other people’s views can only be credibly considered before and not after the decision is made (Lind and Earley 1992), it is further recommended to offer the possibility of giving feedback before the decision is made (Shapiro 1993) to respond to negative information (Davison et al. 2016), or to offset a negative impression due to a lack of social media information, as discussed in the section on bias suppression. While it is not always useful to base decisions on consensus, a so-called *consultative* style of decision making as proposed by Vroom and Yetton (1973) may be useful in screening social media for hiring decisions. According to this concept, the decision-maker makes the final decision, yet it is based on the opinions of others when consulted. As it is advisable to have several people involved in the social media screening process (Davison et al. 2012, 2016), the consultative style should also be applied to the team of reviewers, as well as the representatives of interest groups, who are involved in the screening process to have their views and recommendations heard. In order to solve the missing opportunity of applicants’ exercising “voice”, Slovensky and Ross (2012) discuss the possibility of the recruiter reviewing the social media profile together with applicants, so as to give them the possibility to comment on possible “unprofessional” content directly. However, this practice may still be perceived as unprofessional or voyeuristic, and it may hit the candidate unexpectedly during an interview, if not communicated properly. Therefore, notifying applicants in advance that the hiring manager may review the social media profile during the interview should be considered. Still, it may lead to a candidate’s negative overall perception of the organization by thinking the employer is generally unfair through engaging in social media screening (Slovensky and Ross 2012). Research suggests that for achieving a positive perception of procedural justice, it is not sufficient to give individuals the opportunity of expressing voice, but to give them the impression that the listener actually *considers* their opinions (Tyler 1987; Lind and Earley 1992; Shapiro 1993). In addition to the importance of process control and outcome control, researchers have found the perceived sensitivity and interpersonal qualities of a third party to be influential in procedural justice judgments (Bies et al. 1988; Lind and Tyler 1988; Greenberg 1990a). According to Conlon and Ross (2012), it is part of the voice rule to listen and show appreciation for the other party’s concerns, even if it may contradict one’s own position. It is therefore recommended to be interpersonally attentive in responding to the expressed opinions of applicants. This would include gestures of attention like eye contact, taking notes, listening without preconceived notions, not interrupting the applicant with objections, explicitly attempting to understand what the applicant has said by repeating or asking for clarification, offering help by saying that the applicant’s concerns will be shared with powerful others, and by exhibiting politeness and respect in general (Shapiro 1993). While the sheer opportunity for voice as well as gestures of appreciation are considered helpful in this context, it should be noted that applicants’ perspectives towards social media screening need to be truly considered and *understood* before engaging in it. For this reason, Cook et al. (2020) recommend to quantify applicants’ perceptions of social media screening beforehand. For example, it may be assessed whether they perceive it as an invasion of privacy. A summary of the recommendations to enhance voice in the social media screening process is provided in Table 5.

**Table 5:** Recommendations to enhance voice in social media screening

Recommendations	Selected Literature
• Allow applicants to express voice	Shapiro (1993); Conlon and Ross (2012); Davison et al. (2016)
• Provide the opportunity for two-way communication with applicants during the selection process, *before* the decision is made	Shapiro (1993); Hurrell et al. (2017)
• Actively give applicants the impression of being considered	Tyler (1987); Bies et al. (1988); Lind and Tyler (1988); Greenberg (1990a); Lind and Earley (1992); Shapiro (1993); Conlon and Ross (2012)
• Quantify applicants’ perceptions of social media screening before the screening process	Cook et al. (2020)
• Adopt a consultative style of decision making, also within the pool of raters	Vroom and Yetton (1973); Shapiro (1993)
• Review the social media profiles together with candidates and allow them to comment	Slovensky and Ross (2012)

### Correctability

Acknowledging that even the most well-intentioned and competent decision makers are not free from error, the correctability rule requires that it must be possible to modify and reverse decisions that emerged at different points in the allocative process (Leventhal 1980). The section on accuracy of information has demonstrated that the social media screening process is prone to error in many ways. As discussed earlier, the hiring decision should not be taken by looking at social media in isolation and, as pointed out by Schroeder et al. (2020), social media should not be used to replace traditional selection methods. Rather, they should include a number of steps and sources of information traditionally used in the selection process, as proposed by Landers and Schmidt (2016). It is difficult to reverse a hiring decision once it is made, but the rule of correctability may be applied to social media screening as one step in the selection process. Furthermore, to make the rule of correctability of the allocation process perceptible, Shapiro (1993) recommends explicitly stating that the decision is not yet made or that it is correctable. This way, hiring managers give the impression that the individual’s opinion is taken into consideration and included in the decision-making process (Shapiro 1993), as required by the voice rule. Thus, the rule of correctability can be applied by communicating that the decision is not yet made and still correctable at a particular point of a multiple-step-process. Table 6 provides a summary of these recommendations.

**Table 6:** Recommendations to enable correctability in social media screening

Recommendations	Selected Literature
• Make hiring decisions not based on social media screening alone, but using several steps and sources of information	Landers and Schmidt (2016); Schroeder et al. (2020)
• Communicate that the final decision is not yet made at this point of the selection process	Leventhal (1980); Shapiro (1993)

### Ethicality

The ethicality rule according to Leventhal (1980) requires allocative processes to comply with fundamental moral and ethical values accepted by the individual, who is subject to the allocation process. If such personal standards of ethics and morality are violated, perceived procedural justice is reduced. In addressing this rule, Leventhal (1980) points quite explicitly to cases in which information is collected about potential receivers of rewards. He states that in such cases, methods are deemed unfair according to the ethicality rule if they involve deception or privacy invasion. This may have strong implications for the use of social media screening in practice, as it represents such a process of gathering information about the applicant as a potential receiver of rewards in the form of the job offer. The relationship between privacy and procedural justice has been subject to various empirical studies (Stoughton et al. 2015). For example, Alge (2001) investigated the matter under the conditions of computer surveillance of employees and found a mediating effect of privacy invasions on the relation between procedures (in this case the practice of electronic monitoring) and procedural justice. Chory et al. (2015) found a positive relationship between employee privacy and perceptions of procedural justice. Stoughton et al. (2015) investigated the relationship between perceived invasions of privacy as a result of social media screening and perceived procedural justice. They concluded that privacy perceptions mediate the effects of social media screening on procedural justice views.

By conducting social media assessment, employers risk intruding into areas of privacy not relevant to the application process and thereby intentionally or unintentionally violating privacy laws (Black and Johnson 2012). Especially when accessing highly sensitive and personal information not publicly available, the employer risks a violation of applicants’ privacy, as well as potential lawsuits for invasion of privacy as part of a hiring practice that is not validated (Jeske and Shultz 2016). Clark and Roberts (2010) present an extensive discussion of user privacy in the context of social media screening. They argue that the practice of social media screening attacks individuals’ natural right to have a personal space and that areas of privacy should be protected from employer use. Following their argument, applicants regard social media activities as part of their private, non-work-related life. Therefore, Clark and Roberts (2010) conclude that it is beneficial for society to protect this boundary between professional and private life. Similarly, Stoughton et al. (2015) note that such a transgression of boundaries poses a basic issue of fairness, since users post content on their social media websites for private purposes, while employers use this information for professional purposes. Applicants thus tend to perceive social media screening as unfair because they view their social media usage as part of their non-work environment and favour hiring procedures that are job-related. The study conducted by Hurrell et al. (2017) on procedural justice effects of social media screening on Generation Y supports this view, as they found younger study participants to be particularly alert towards the blurring work-life boundary through social media screening. Subsequently, according to Thomas et al. (2015), privacy advocates contend that social media communication as a form of private conversations is at danger if the possibility exists that employers could judge individuals based on their social interaction online. Clark and Roberts (2010) warn that under the pressure to conform, people will modify what they post, which may lead to an overall chilling effect on this form of communication and a society of “mediocre persons.” According to the findings of Alge (2001) and Stoughton et al. (2015), a perceived breach of privacy may even result in an applicant’s self-devaluation, as personal identity is strongly linked with perceptions of privacy. A transgression of the boundary between an individual’s private and public self-representation may therefore result in a lack of control over one’s public persona.

However, the question whether employers do indeed commit breaches of privacy by engaging in social media screening is not an easy one to answer. According to Brandenburg (2008), for a true violation to happen, the person must have a reasonable expectation of privacy in the first place. Although many employees tend to believe that they have a right to privacy on social media outside their workplace (Chauhan et al. 2013), the question as to whether or not users should have an expectation of privacy is highly debated (Brandenburg 2008; Elzweig and Peeples 2009). Privacy advocates and other commentators voice their criticism of the practice of screening social media in selection, but proponents argue that information on social media is public and that employers have a right to access this information in their duty to protect the organization (Thomas et al. 2015). Similarly, some commentators claim that the expectation of privacy is given up once information is posted on social media (Chauhan et al. 2013). The expectation of privacy may also strongly depend on the privacy conditions and settings of the operator of a social network (Elzweig and Peeples 2009), which generally differ from platform to platform, making it difficult to assess privacy expectations (Drake 2016). Moreover, the operators of social networks can change the privacy terms at any time, thus reducing employee privacy by including phrases like “we reserve the right, at our sole discretion, to change, modify, add, or delete portions of the terms of use at any time without further notice” (Kaupins and Park 2011, p. 92). There is general agreement that someone who does not use privacy settings in social networks cannot have an expectation of privacy (Elzweig and Peeples 2009), and someone who at least attempts to protect their privacy through the use of privacy settings deserves more recognition of privacy than someone who does not make such an attempt. However, the question of procedural fairness is concerned with *perceived* breaches of privacy, as the ethicality rule of Leventhal (1980) deals with the violation of the ethical standards of the person who is subject to the decision process. Even if the procedure was fair from a legal standpoint and the person did not have a legal expectation of privacy, a perceived breach of privacy may still lead to decreased procedural fairness from that person’s point of view. After all, a perceived breach of privacy may also have negative effects on the company engaging in social media screening, such as a decrease in the company’s attractiveness and a higher probability of disgruntled applicants suing them. Thereby, harm may be caused in the form of legal costs, declines in customer loyalty, shareholder value, and overall damage to the organization’s reputation (Stoughton et al. 2015). Therefore, it is to be concluded that the company should have at least some interest in avoiding such perceived privacy violations beyond mere obedience to the law.

To address the ethical dilemma of obtaining the necessary information for putting the right person into the job without violating fundamental moral and ethical standards, a number of recommendations can be drawn from the literature. These recommendations are summarized in Table 7. To avoid the risk of getting involved in privacy infringement or discrimination, it is recommended to check only publicly available sites, especially after personal interviews have already taken place, so basic personal traits can already be known (Lam 2016). Several authors discuss the possibility of informing the candidate beforehand (Slovensky and Ross 2012; Hoek et al. 2016; Lam 2016) or asking for written permission (Slovensky and Ross 2012). Yet, a word of caution remains. Organizations may, nevertheless, receive negative reactions in the process of notifying applicants and obtaining permission to screen social media profiles (Davison et al. 2016), or applicants may clean up their social media profiles and try to make the profile look more favourable (Black and Johnson 2012; Slovensky and Ross 2012; Davison et al. 2016). Asking for candidates’ consent should also be handled with care considering the applicants’ situation of dependency in search for a job: Any refusal to agree to social media screening may be perceived negatively by the employer and may therefore lead to bias, since applicants are not free from coercion in this situation (Lam 2016).

**Table 7:** Recommendations to enhance the ethicality of social media screening

Recommendations	Selected Literature
• Check only publicly available sites	Lam (2016)
• Inform candidates beforehand that social media screening is used in the selection process	Slovensky and Ross (2012); Hoek et al. (2016); Lam (2016)
• Obtain written permission to view candidates’ social media profiles	Slovensky and Ross (2012); Davison et al. (2016)

## Summary of Recommendations

**Fig. 1:**
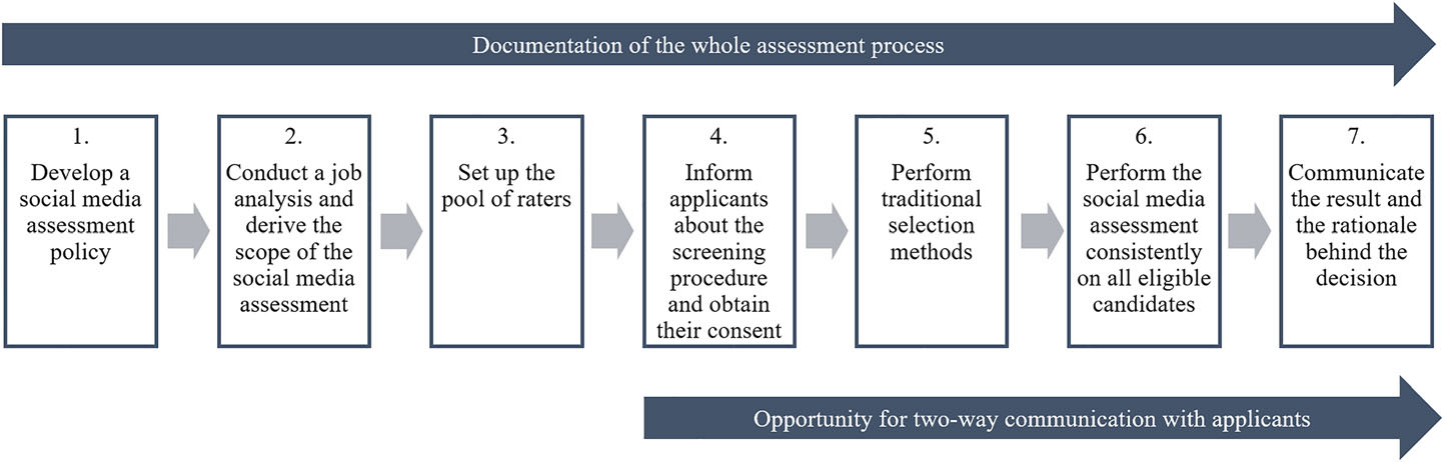
Proposed procedure in social media screening

As the analysis of the extended rules of procedural justice of Leventhal (1980) in relation to social media screening has demonstrated, there are several points at which the procedural justice of social media screening is rather questionable. The discussion has shown that, despite all benefits, social media screening in itself cannot be considered a procedurally just assessment tool. These findings confirm the results from previous research suggesting that the use of social media screening has a negative impact on applicants’ perceptions of procedural justice (Stoughton et al. 2015; Stoughton 2016; Hurrell et al. 2017). Following Davison et al.’s (2016) primary recommendation not to use social media screening at all when in doubt about the legal and ethical implications of this practice, an organization should refrain from the use of social media screening if no efforts can be made to ensure fairness in the process. However, the potential benefits should not be neglected. The discussion has shown social media screening to have the potential of being a helpful contemporary tool in identifying the right job candidate and avoiding irresponsible behaviour and negligent hiring, as long as it is applied with care. The recent COVID-19 crisis highlights that companies need to be able to respond to new challenges through the effective use of digital technologies (Guo et al. 2020). As digital communication continues to challenge recruitment and selection practices (Holland and Jeske 2017), social media screening should be evaluated and used in the sense of a responsible HR practice that benefits both the company and its potential employees in the long term. Consistent with Kluemper et al.’s (2015) conclusion about the fairness of social media screening, stating that “applicants may not always react negatively to SNW [Social Network Website] screening, depending on the screening approach” (p. 74), research-based efforts need to be made to construct the process in a fair manner. Thus, the recommendations pertaining to each of the rules discussed are summarized in Fig. 1. Thereby, the first key recommendation that can be drawn from the discussion is to standardize the social media screening process and to assign clear responsibilities among the people conducting the screening and making the employment decision. The second key recommendation is to ensure that the information obtained through social media screening is relevant for the position that needs to be filled, which can be achieved through a job analysis. Thirdly, it is important to allow for an adequate degree of transparency and two-way communication with applicants, including the information that social media profiles may be checked in the selection process.

## Conclusion

The aim of this paper was to discuss whether social media screening can be considered a procedurally fair tool for selecting applicants in the hiring process. The discussion showed that it holds many benefits for employers, as it provides them with access to information not easily obtained through traditional assessment methods at relatively low costs. This allows companies of all sizes to identify the right person for a job by making inferences about candidates’ character, performance, and fit with the organization, thereby mitigating the risk for the company’s stakeholders and fostering a positive work environment. Companies that bear a particular responsibility towards their stakeholders can eliminate potentially harmful or even criminal candidates from the hiring process. However, the discussion shows that, according to Leventhal’s (1980) extended rules of procedural justice, social media screening bears manifold risks of violating procedural justice. Despite at least partly objecting to Clark and Roberts’ (2010) position deeming social media screening an unethical practice, the discussion has demonstrated that without the necessary precautions, social media screening cannot be considered procedurally fair, as it opens up far too many opportunities for inaccurate or job-irrelevant information, lack of transparency, discrimination, recruiter biases, and privacy violations. It is therefore essential to follow some basic guidelines to address the aforementioned issues and to enhance procedural justice. While this paper represents an attempt to derive such guidelines from the literature, not all issues will be resolved, as the practice differs from company to company, and the ethical values of the applicants differ among individuals and cultural contexts. It is therefore necessary to carry out an evidence-based study for policy development, which takes into consideration the concerns of individual users as well as companies’ requirements for performing social media screening. In particular, the review of the work of Schroeder et al. (2020) suggests that different structuring methods of social media screening may lead to varying results. Therefore, future empirical research on the effectiveness of different methods of structuring the process of social media screening may provide further insights for practitioners.

## References

[CR1] Adams, J. S. (1965). Inequity in social exchange. In L. Berkowitz (Ed.), *Advances in experimental social psychology (pp. 267–299)*. Elsevier. 10.1016/S0065-2601(08)60108-2.

[CR2] Aguado, D., Rico, R., Rubio, V. J., & Fernández, L. (2016). Applicant reactions to social network web use in personnel selection and assessment. *Revista de Psicología del Trabajo y de las Organizaciones, 32*(3), 183–190. 10.1016/j.rpto.2016.09.001.

[CR3] Alder, G. S., & Gilbert, J. (2006). Achieving ethics and fairness in hiring: Going beyond the law. *Journal of Business Ethics, 68*(4), 449–464. 10.1007/s10551-006-9039-z.

[CR4] Alexander, E. C., Mader, D. R. D., & Mader, F. H. (2019). Using social media during the hiring process: A comparison between recruiters and job seekers. *Journal of Global Scholars of Marketing Science, 29*(1), 78–87. 10.1080/21639159.2018.1552530.

[CR5] Alge, B. J. (2001). Effects of computer surveillance on perceptions of privacy and procedural justice. *Journal of Applied Psychology, 86*(4), 797–804. 10.1037//0021-9010.86.4.797. 11519663 10.1037/0021-9010.86.4.797

[CR6] Bapuji, H., De Bakker, F. G. A., Brown, J. A., Higgins, C., Rehbein, K., & Spicer, A. (2020). Business and society research in times of the Corona crisis. *Business & Society, 59*(6), 1067–1078. 10.1177/0007650320921172.

[CR7] Becton, J. B., Walker, H. J., Gilstrap, J. B., & Schwager, P. H. (2019). Social media snooping on job applicants. *Personnel Review, 48*(5), 1261–1280. 10.1108/PR-09-2017-0278.

[CR8] Bentley, E. D. (2013). The pitfalls of using social media screening for job applicants. *ABA Journal of Labor & Employment Law, 29*(1), 1–13.

[CR9] Berkelaar, B. L. (2014). Cybervetting, online information, and personnel selection. *Management Communication Quarterly, 28*(4), 479–506. 10.1177/0893318914541966.

[CR10] Berkelaar, B. L. (2017). Different ways new information technologies influence conventional organizational practices and employment relationships: The case of cybervetting for personnel selection. *Human Relations, 70*(9), 1115–1140. 10.1177/0018726716686400.

[CR11] Berkelaar, B. L., & Buzzanell, P. M. (2014). Online employment screening and digital career capital. *Management Communication Quarterly, 29*(1), 84–113. 10.1177/0893318914554657.

[CR12] Bertrand, M., & Duflo, E. (2017). Field experiments on discrimination. In a. V. Banerjee & E. Duflo (Eds.), *Handbook of economic field experiments* (pp. 309–393). 10.1016/bs.hefe.2016.08.004.

[CR13] Bies, R. J., & Moag, J. F. (1986). Interactional justice: Communication criteria of fairness. In R. J. Lewicki, B. H. Sheppard, & M. H. Bazerman (Eds.), *Research on negotiations in organizations* (pp. 43–55). JAI Press.

[CR14] Bies, R. J., Shapiro, D. L., & Cummings, L. L. (1988). Causal accounts and managing organizational conflict. *Communication Research, 15*(4), 381–399. 10.1177/009365088015004003.

[CR15] Black, S. L., & Johnson, A. F. (2012). Employers’ use of social networking sites in the selection process. *The Journal of Social Media in Society, 1*(1), 7–28.

[CR16] Black, S. L., Johnson, A. F., Takach, S. E., & Stone, D. L. (2012). Factors affecting applicants’ reactions to the collection of data in social network websites. *Academy of Management Proceedings, 2012*(1), 11742.

[CR17] Black, S. L., Stone, D. L., & Johnson, A. F. (2015). Use of social networking websites on applicants’ privacy. *Employee Responsibilities and Rights Journal, 27*(2), 115–159. 10.1007/s10672-014-9245-2.

[CR18] Brandenburg, C. (2008). The newest way to screen job applicants: A social networker’s nightmare. *Federal Communications Law Journal, 60*(3), 597–626.

[CR19] Brebels, L., De Cremer, D., Van Dijke, M., & Van Hiel, A. (2010). Fairness as social responsibility: A moral self-regulation account of procedural justice enactment. *British Journal of Management, 22*, 47–58. 10.1111/j.1467-8551.2010.00715.x.

[CR20] Brown, V. R., & Vaughn, E. D. (2011). The writing on the (Facebook) wall: The use of social networking sites in hiring decisions. *Journal of Business and Psychology, 26*(2), 219–225. 10.1007/s10869-011-9221-x.

[CR21] Buchanan, A., & Mathieu, D. (2013). Philosophy and justice. In R. L. Cohen (Ed.), *Justice: Views from the social sciences* (pp. 11–45). New York: Springer. 10.1007/978-1-4899-3511-3_2.

[CR22] Caligiuri, P., De Cieri, H., Minbaeva, D., Verbeke, A., & Zimmermann, A. (2020). International HRM insights for navigating the COVID-19 pandemic: Implications for future research and practice. *Journal of International Business Studies, 51*(5), 1–17. 10.1057/s41267-020-00335-9. 10.1057/s41267-020-00335-9 PMC7266413 32836500

[CR23] Chauhan, R., Buckley, R., & Harvey, M. (2013). Facebook and personnel selection: What’s the big deal? *Organizational Dynamics, 42*(2), 126–134. 10.1016/j.orgdyn.2013.03.006.

[CR24] Chory, R. M., Vela, L. E., & Avtgis, T. A. (2015). Organizational surveillance of computer-mediated workplace communication: Employee privacy concerns and responses. *Employee Responsibilities and Rights Journal, 28*(1), 23–43. 10.1007/s10672-015-9267-4.

[CR25] Clark, L. A., & Roberts, S. J. (2010). Employer’s use of social networking sites: A socially irresponsible practice. *Journal of Business Ethics, 95*(4), 507–525. 10.1007/s10551-010-0436-y.

[CR26] Cohen, R. L. (1987). Distributive justice: Theory and research. *Social Justice Research, 1*(1), 19–40. 10.1007/BF01049382.

[CR27] Colquitt, J. A. (2001). On the dimensionality of organizational justice: A construct validation of a measure. *The Journal of Applied Psychology, 86*(3), 386–400. 10.1037/0021-9010.86.3.386. 11419799 10.1037/0021-9010.86.3.386

[CR28] Colquitt, J. A., Conlon, D. E., Wesson, M. J., Porter, C. O., & Ng, K. Y. (2001). Justice at the millennium: A meta-analytic review of 25 years of organizational justice research. *Journal of Applied Psychology, 86*(3), 425–445. 10.1037//0021-9010.86.3.425. 11419803 10.1037/0021-9010.86.3.425

[CR29] Conlon, D., & Ross, W. H. (2012). The effect of perceived/felt (in)justice on cooperativeness: Implications for negotiators as “justice-enhancing communicators” in an era of social networking. In B. Goldman & D. L. Shapiro (Eds.), *The psychology of negotiations in the 21*^*st*^*century workplace: New challenges and new solutions* (pp. 17–43). Routledge.

[CR30] Connerley, M. L., Arvey, R. D., & Bernardy, C. J. (2001). Criminal background checks for prospective and current employees: Current practices among municipal agencies. *Public Personnel Management, 30*(2), 173–183. 10.1177/009102600103000204.

[CR31] Cook, R., Jones-Chick, R., Roulin, N., & O'Rourke, K. (2020). Job seekers' attitudes toward cybervetting: Scale development, validation, and platform comparison. *International Journal of Selection and Assessment, 28*(4), 383–398. 10.1111/ijsa.12300.

[CR32] Cropanzano, R., & Greenberg, J. (1997). Progress in organizational justice: Tunneling through the maze. *International Review of Industrial and Organizational Psychology, 12*, 317–372.

[CR33] Cropanzano, R., & Ambrose, M. L. (2002). Procedural and distributive justice are more similar than you think: A monistic perspective and a research agenda. In J. Greenberg & R. Cropanzano (Eds.), *Advances in organizational justice* (pp. 119–151). Stanford University Press.

[CR34] Cropanzano, R., & Randall, M. L. (1993). Injustice and work behaviour: A historical review. In R. Cropanzano (Ed.), *Justice in the workplace* (pp. 3–20). L. Erlbaum Associates.

[CR35] Davis, D. C. (2007). MySpace isn’t your space: Expanding the fair credit reporting act to ensure accountability and fairness in employer searches of online social networking services. *Kansas Journal of Law & Public Policy, 16*(2), 237–256.

[CR36] Davison, H. K., Bing, M. N., Kluemper, D. H., & Roth, P. L. (2016). Social media as a personnel selection and hiring resource: Reservations and recommendations. In R. N. Landers & G. B. Schmidt (Eds.), *Social media in employee selection and recruitment: Theory, practice, and current challenges* (pp. 15–42). Springer International Publishing.

[CR37] Davison, H. K., Maraist, C., & Bing, M. N. (2011). Friend or foe? The promise and pitfalls of using social networking sites for HR decisions. *Journal of Business and Psychology, 26*(2), 153–159. 10.1007/s10869-011-9215-8.

[CR38] Davison, H. K., Maraist, C. C., Hamilton, R. H., & Bing, M. N. (2012). To screen or not to screen? Using the internet for selection decisions. *Employee Responsibilities and Rights Journal, 24*(1), 1–21. 10.1007/s10672-011-9178-y.

[CR39] Demuijnck, G. (2009). Non-discrimination in human resources management as a moral obligation. *Journal of Business Ethics, 88*(1), 83–101. 10.1007/s10551-009-0100-6.

[CR40] Drake, J. R. (2016). Asking for Facebook logins: An egoist case for privacy. *Journal of Business Ethics, 139*(3), 429–441. 10.1007/s10551-015-2586-4.

[CR41] Drake, J. R., Hall, D., Becton, B., & Posey, C. (2016). Job applicants’ information privacy protection responses: Using social media for candidate screening. *Transactions on Human-Computer Interaction, 8*(4), 160–184.

[CR42] Ege, R., & Igersheim, H. (2010). Rawls’s justice theory and its relations to the concept of merit goods. *The European Journal of the History of Economic Thought, 17*(4), 1001–1030. 10.1080/09672567.2010.482999.

[CR43] Elzweig, B., & Peeples, D. K. (2009). Using social networking web sites in hiring and retention decisions. *SAM Advanced Management Journal, 74*(4), 27–35.

[CR44] Evuleocha, S. U., & Ugbah, S. D. (2018). Profiling: The efficacy of using social networking sites for job screening. *Journal of Employment Counseling, 55*(2), 48–57. 10.1002/joec.12074.

[CR45] Flyverbom, M., Deibert, R., & Matten, D. (2019). The governance of digital technology, big data, and the internet: New roles and responsibilities for business. *Business & Society, 58*(1), 3–19. 10.1177/0007650317727540.

[CR46] Folger, R. (1977). Distributive and procedural justice: Combined impact of voice and improvement on experienced inequity. *Journal of Personality and Social Psychology, 35*(2), 108–119. 10.1037/0022-3514.35.2.108.

[CR47] Folger, R., & Konovsky, M. A. (1989). Effects of procedural and distributive justice on reactions to pay raise decisions. *Academy of Management Journal, 32*(1), 115–130. 10.5465/256422.

[CR48] Gilliland, S. W. (1993). The perceived fairness of selection systems: An organizational justice perspective. *Academy of Management Review, 18*(4), 694–734. 10.5465/amr.1993.9402210155.

[CR49] Gilliland, S. W. (1994). Effects of procedural and distributive justice on reactions to a selection system. *Journal of Applied Psychology, 79*(5), 691–701. 10.1037/0021-9010.79.5.691.

[CR50] Goffman, E. (1961). *Encounters: Two studies in the sociology of interaction*. Bobbs-Merrill.

[CR51] Goffman, E. (2005). Role Distance. In D. Brissett & C. Edgley (Eds.), *Life as theater: A dramaturgical sourcebook* (pp. 101–111). Aldine Transaction.

[CR52] Greenberg, J. (1987). A taxonomy of organizational justice theories. *Academy of Management Review, 12*(1), 9–22. 10.5465/amr.1987.4306437.

[CR53] Greenberg, J. (1990a). Employee theft as a reaction to underpayment inequity: The hidden cost of pay cuts. *Journal of Applied Psychology, 75*(5), 561–568. 10.1037/0021-9010.75.5.561.

[CR54] Greenberg, J. (1990b). Organizational justice: Yesterday, today, and tomorrow. *Journal of Management, 16*(2), 399–432. 10.1177/014920639001600208.

[CR55] Greenberg, J., & Bies, R. J. (1992). Establishing the role of empirical studies of organizational justice in philosophical inquiries into business ethics. *Journal of Business Ethics, 11*(5), 433–444. 10.1007/BF00870555.

[CR56] Greenberg, J., & Cohen, R. L. (2014). The justice concept in social psychology. In J. Greenberg & R. L. Cohen (Eds.), *Equity and justice in social behaviour* (pp. 1–42). Academic Press.

[CR57] Greenberg, J., & Folger, R. (1983). Procedural justice, participation, and the fair process effect in groups and organizations. In P. B. Paulus (Ed.), *Basic group processes. Springer series in social psychology* (pp. 235–256). New York: Springer. 10.1007/978-1-4612-5578-9_10.

[CR58] Guo, H., Yang, Z., Huang, R., & Guo, A. (2020). The digitalization and public crisis responses of small and medium enterprises: Implications from a COVID-19 survey. *Frontiers of Business Research in China, 14*(1), 1–25. 10.1186/s11782-020-00087-1.

[CR59] Hartman, S. J., Yrle, A. C., & Galle, W. P. (1999). Procedural and distributive justice: Examining equity in a university setting. *Journal of Business Ethics, 20*(4), 337–352. 10.1023/A:1006102216883.

[CR60] Hausknecht, J. P., Day, D. V., & Thomas, S. C. (2004). Applicant reactions to selection procedures: An updated model and meta-analysis. *Personnel Psychology, 57*(3), 639–683. 10.1111/j.1744-6570.2004.00003.x.

[CR61] Henderson, K. E. (2019). They posted what? Recruiter use of social media for selection. *Organizational Dynamics, 48*(4), 100663. 10.1016/j.orgdyn.2018.05.005.

[CR62] Hoek, J., O’Kane, P., & McCracken, M. (2016). Publishing personal information online. *Personnel Review, 45*(1), 67–83. 10.1108/PR-05-2014-0099.

[CR63] Holland, P., & Jeske, D. (2017). Changing role of social media at work: Implications for recruitment and selection. In T. Bondarouk, J. M. Huub, & E. Parry (Eds.), *Electronic HRM in the smart era (pp. 115–129)*. Emerald Publishing Limited.

[CR64] Hunter, J. E., & Hunter, R. F. (1984). Validity and utility of alternative predictors of job performance. *Psychological Bulletin, 96*(1), 72–98. 10.1037/0033-2909.96.1.72.

[CR65] Hurrell, S. A., Scholarios, D., & Richards, J. (2017). ‘The kids are alert’: Generation Y responses to employer use and monitoring of social networking sites. *New Technology, Work and Employment, 32*(1), 64–83. 10.1111/ntwe.12085.

[CR66] Jeske, D., & Shultz, K. S. (2016). Using social media content for screening in recruitment and selection: Pros and cons. *Work, Employment and Society, 30*(3), 535–546. 10.1177/0950017015613746.

[CR67] Jeske, D., & Shultz, K. S. (2019). Social media screening and content effects: Implications for job applicant reactions. *International Journal of Manpower, 40*(1), 73–86. 10.1108/IJM-06-2017-0138.

[CR68] Jones, C., & Behling, S. (2010). Unchartered waters: Using social networks in hiring decisions. *Issues in Information Systems, 11*(1), 589–595.

[CR69] Karl, K., Peluchette, J., & Schlaegel, C. (2010). Who’s posting Facebook faux pas? A cross-cultural examination of personality differences. *International Journal of Selection and Assessment, 18*(2), 174–186. 10.1111/j.1468-2389.2010.00499.x.

[CR70] Kaupins, G., & Park, S. (2011). Legal and ethical implications of corporate social networks. *Employee Responsibilities and Rights Journal, 23*(2), 83–99. 10.1007/s10672-010-9149-8.

[CR71] Kittay, E. F. (2018). Human dependency and Rawlsian equality. In D. T. Meyers (Ed.), *Feminists rethink the self* (pp. 219–266). Taylor & Francis.

[CR72] Kluemper, D. H. (2013). Social network screening: Pitfalls, possibilities, and parallels in employment selection. *Social Media in Human Resources Management (Advanced Series in Management), 12*, 1–21. 10.1108/S1877-6361(2013)0000012005.

[CR73] Kluemper, D. H., Davison, H. K., Cao, X., & Wu, B. (2015). Social networking websites and personnel selection: A call for academic research. In I. Nikolaou & J. K. Oostrom (Eds.), *Employee recruitment, selection, and assessment: Contemporary issues for theory and practice* (pp. 61–79). Taylor and Francis.

[CR74] Kluemper, D. H., & Rosen, P. A. (2009). Future employment selection methods: Evaluating social networking web sites. *Journal of Managerial Psychology, 24*(6), 567–580. 10.1108/02683940910974134.

[CR75] Kluemper, D. H., Rosen, P. A., & Mossholder, K. W. (2012). Social networking websites, personality ratings, and the organizational context: More than meets the eye? *Journal of Applied Social Psychology, 42*(5), 1143–1172. 10.1111/j.1559-1816.2011.00881.x.

[CR76] Kohlberg, L. (1973). The claim to moral adequacy of a highest stage of moral judgement. *Journal of Psychology, 70*(18), 630–646.

[CR77] Kohlberg, L. (1981). *Essays on moral development*. Harper & Row.

[CR78] Konow, J. (2003). Which is the fairest one of all? A positive analysis of justice theories. *Journal of Economic Literature, 41*(4), 1188–1239. 10.1257/002205103771800013.

[CR79] Lam, H. (2016). Social media dilemmas in the employment context. *Employee Relations, 38*(3), 420–437. 10.1108/ER-04-2015-0072.

[CR80] Landers, R. N., & Schmidt, G. B. (2016). Social media in employee selection and recruitment: Current knowledge, unanswered questions, and future directions. In R. N. Landers & G. B. Schmidt (Eds.), *Social media in employee selection and recruitment: Theory, practice, and current challenges* (pp. 343–367). Springer International Publishing.

[CR81] Lee, A. R., Son, S. M., & Kim, K. K. (2016). Information and communication technology overload and social networking service fatigue: A stress perspective. *Computers in Human Behavior, 55*, 51–61. 10.1016/j.chb.2015.08.011.

[CR82] Leventhal, G. S. (1980). What should be done with equity theory? In K. J. Gergen, M. S. Greenberg, & R. H. Willis (Eds.), *Social exchange: Advances in theory and research* (pp. 27–55). Springer. 10.1007/978-1-4613-3087-5_2.

[CR83] Lind, E. A., & Earley, P. C. (1992). Procedural justice and culture. *International Journal of Psychology, 27*(2), 227–242. 10.1080/00207599208246877.

[CR84] Lind, E. A., & Tyler, T. R. (1988). *The social psychology of procedural justice*. New York: Plenum Press.

[CR85] Loi, R., Lam, L. W., & Chan, K. W. (2012). Coping with job insecurity: The role of procedural justice, ethical leadership and power distance orientation. *Journal of Business Ethics, 108*(3), 361–372. 10.1007/s10551-011-1095-3.

[CR86] Maier, C., Laumer, S., Weinert, C., & Weitzel, T. (2015). The effects of technostress and switching stress on discontinued use of social networking services: A study of Facebook use. *Information Systems Journal, 25*(3), 275–308. 10.1111/isj.12068.

[CR87] Manant, M., Pajak, S., & Soulié, N. (2019). Can social media lead to labor market discrimination? Evidence from a field experiment. *Journal of Economics & Management Strategy, 28*(2), 225–246. 10.1111/jems.12291.

[CR88] McDonald, P., & Thompson, P. (2016). Social media(tion) and the reshaping of public/private boundaries in employment relations. *International Journal of Management Reviews, 18*(1), 69–84. 10.1111/ijmr.12061.

[CR89] McFarlin, D. B., & Sweeney, P. D. (1992). Research notes: Distributive and procedural justice as predictors of satisfaction with personal and organizational outcomes. *Academy of Management Journal, 35*(3), 626–637. 10.5465/256489.

[CR90] McGowan, R. (1990). Justice: The root of American business ideology and ethics. *Journal of Business Ethics, 9*(11), 891–901. 10.1007/BF00382912.

[CR91] Nikolaou, I. (2014). Social networking web sites in job search and employee recruitment. *International Journal of Selection and Assessment, 22*(2), 179–189. 10.1111/ijsa.12067.

[CR92] Novelli, L., Kirkman, B. L., & Shapiro, D. L. (1995). Effective implementation of organizational change: An organizational justice perspective. *Journal of Organizational Behavior (1986-1998)*, 15–47.

[CR93] Nowakowski, J. M., & Conlon, D. E. (2005). Organizational justice: Looking back, looking forward. *International Journal of Conflict Management, 16*(1), 4–29. 10.1108/eb022921.

[CR94] Peluchette, J., & Karl, K. (2009). Examining students’ intended image on Facebook: “What were they thinking?!”. *Journal of Education for Business, 85*(1), 30–37. 10.1080/08832320903217606.

[CR95] Ployhart, R. E., & Ryan, A. M. (1997). Toward an explanation of applicant reactions: An examination of organizational justice and attribution frameworks. *Organizational Behavior and Human Decision Processes, 72*(3), 308–335. 10.1006/obhd.1997.2742. 9606169 10.1006/obhd.1997.2742

[CR96] Ployhart, R. E., & Ryan, A. M. (1998). Applicants’ reactions to the fairness of selection procedures: The effects of positive rule violations and time of measurement. *Journal of Applied Psychology, 83*(1), 3–16. 10.1037/0021-9010.83.1.3. 9494438 10.1037/0021-9010.83.1.3

[CR97] Rawls, J. (1971). *A theory of justice*. Harvard University Press.

[CR98] Rosen, P. A., Solomon, S. J., McLarty, B. D., Esken, C. A., & Taylor, E. C. (2018). The use of twitter profiles to assess personality and hireability. *Journal of Managerial Issues, 30*(2), 256–272.

[CR99] Roth, P. L., Bobko, P., Van Iddekinge, C. H., & Thatcher, J. B. (2016). Social media in employee-selection-related decisions: A research agenda for unchartered territory. *Journal of Management, 42*(1), 269–298. 10.1177/0149206313503018.

[CR100] Schroeder, A. N., Odd, K., & Whitaker, J. H. (2020). Agree to disagree: Examining the psychometrics of cybervetting. *Journal of Managerial Psychology, 35*(5), 435–450. 10.1108/JMP-09-2018-0420.

[CR101] Schuler, H. (1993). Social validity of selection situations: A concept and some empirical results. In H. Schuler, J. L. Farr, & M. Smith (Eds.), *Personnel selection and assessment: Individual and organizational perspectives* (pp. 11–26). Lawrence Erlbaum Associates.

[CR102] Scott, G. G., Sinclair, J., Short, E., & Bruce, G. (2014). It’s not what you say, it’s how you say it: Language use on Facebook impacts employability but not attractiveness. *Cyberpsychology, Behavior and Social Networking, 17*(8), 562–566. 10.1089/cyber.2013.0584. 24949532 10.1089/cyber.2013.0584

[CR103] Shapiro, D. L. (1993). Reconciling theoretical differences among procedural justice researchers by re-evaluating what it means to have one’s views “considered”: Implications for third-party managers. In R. Cropanzano (Ed.), *Justice in the workplace* (pp. 51–78). Lawrence Erlbaum Associates.

[CR104] Sheppard, B. H., Lewicki, R. J., & Minton, J. W. (1992). *Organizational justice: The search for fairness in the workplace*. Lexington Books.

[CR105] Simola, S. (2003). Ethics of justice and care in corporate crisis management. *Journal of Business Ethics, 46*(4), 51–361. 10.1023/A:1025607928196.

[CR106] Singer, M. (1993). *Fairness in personnel selection: An organizational justice perspective*. Avebury.

[CR107] Singer, M. S. (2000). Ethical and fair work behaviour: A normative-empirical dialogue concerning ethics and justice. *Journal of Business Ethics, 28*(3), 187–209. 10.1023/A:1006299811213.

[CR108] Slovensky, R., & Ross, W. H. (2012). Should human resource managers use social media to screen job applicants? Managerial and legal issues in the USA. *Info, 14*(1), 55–69. 10.1108/14636691211196941.

[CR109] Smither, J. W., Reilly, R. R., Millsap, R. E., Pearlman, K., & Stoffey, R. W. (1993). Applicant reactions to selection procedures. *Personnel Psychology, 46*(1), 49–76. 10.1111/j.1744-6570.1993.tb00867.x.

[CR110] Stephens, C. U., & Cobb, A. T. (1999). A Habermasian approach to justice in organizational change: Synthesizing the technical and philosophical perspectives. *Journal of Organizational Change Management, 12*(1), 21–34. 10.1108/09534819910255298.

[CR111] Stoughton, J. W., Thompson, L. F., & Meade, A. W. (2015). Examining applicant reactions to the use of social networking websites in pre-employment screening. *Journal of Business and Psychology, 30*(1), 73–88. 10.1007/s10869-013-9333-6.

[CR112] Stoughton, J. W. (2016). Applicant reactions to social media in selection: Early returns and future directions. In R. Landers & G. Schmidt (Eds.), *Social media in employee selection and recruitment* (pp. 249–263). Cham: Springer. 10.1007/978-3-319-29989-1_12.

[CR113] Tews, M. J., Stafford, K., & Kudler, E. P. (2020). The effects of negative content in social networking profiles on perceptions of employment suitability. *International Journal of Selection and Assessment, 28*(1), 17–30. 10.1111/ijsa.12277.

[CR114] Thibaut, J., & Walker, L. (1975). *Procedural justice: A psychological analysis*. Lawrence Erlbaum Associates.

[CR115] Thomas, S. L., Rothschild, P. C., & Donegan, C. (2015). Social networking, management responsibilities, and employee rights: The evolving role of social networking in employment decisions. *Employee Responsibilities and Rights Journal, 27*(4), 307–323. 10.1007/s10672-014-9250-5.

[CR116] Tyler, T. R. (1987). Conditions leading to value-expressive effects in judgments of procedural justice: A test of four models. *Journal of Personality and Social Psychology, 52*(2), 333–344. 10.1037/0022-3514.52.2.333.

[CR117] Van den Bos, K., Vermunt, R., & Wilke, H. A. M. (1997). Procedural and distributive justice: What is fair depends more on what comes first than on what comes next. *Journal of Personality and Social Psychology, 72*(1), 95–104. 10.1037/0022-3514.72.1.95.

[CR118] Vermunt, R., & Steensma, H. (2001). Stress and justice in organizations: An exploration into justice processes with the aim to find mechanisms to reduce stress. In R. Cropanzano (Ed.), *Justice in the workplace: From theory to practice* (pp. 27–48). Lawrence Erlbaum Associates.

[CR119] Vroom, V. H., & Yetton, P. W. (1973). *Leadership and decision-making*. University of Pittsburgh Press.

[CR120] Wade, J., Roth, P. L., Thatcher, J. B., & Dinger, M. (2020). Social media and selection: Political issue similarity, liking, and the moderating effect of social media platform. *MIS Quarterly, 44*(3), 1301–1357. 10.25300/MISQ/2020/14119.

[CR121] Wang, J., Zheng, B., Liu, H., & Yu, L. (2021). A two-factor theoretical model of social media discontinuance: Role of regret, inertia, and their antecedents. *Information Technology & People, 34*(1), 1–24. 10.1108/ITP-10-2018-0483.

[CR122] Weaver, G. R., & Conlon, D. E. (2003). Explaining façades of choice: Timing, justice effects, and behavioral outcomes. *Journal of Applied Social Psychology, 33*(11), 2217–2243. 10.1111/j.1559-1816.2003.tb01882.x.

[CR123] Webster, J., & Watson, R. T. (2002). Analyzing the past to prepare for the future: Writing a literature review. Guest editorial. *MIS Quarterly, 26*(2), xiii–xxiii.

[CR124] Woska, W. J. (2007). Legal issues for HR professionals: Reference checking/background investigations. *Public Personnel Management, 36*(1), 79–89. 10.1177/009102600703600106.

[CR125] Xiao, L., Pan, T., Mou, J., & Huang, L. (2020). Understanding determinants of social networking service fatigue: An interpretive structural modeling approach. *Information Technology & People (ahead-of-print)., ahead-of-print*. 10.1108/ITP-04-2020-0169.

[CR126] Xu, A. J., Loi, R., & Ngo, H. Y. (2016). Ethical leadership behavior and employee justice perceptions: The mediating role of trust in organization. *Journal of Business Ethics, 134*(3), 493–504. 10.1007/s10551-014-2457-4.

[CR127] Zapata-Phelan, C. P., Colquitt, J. A., Scott, B. A., & Livingston, B. (2009). Procedural justice, interactional justice, and task performance: The mediating role of intrinsic motivation. *Organizational Behavior and Human Decision Processes, 108*(1), 93–105. 10.1016/j.obhdp.2008.08.001.

[CR128] Zhang, S., Zhao, L., Lu, Y., & Yang, J. (2016). Do you get tired of socializing? An empirical explanation of discontinuous usage behaviour in social network services. *Information & Management, 53*(7), 904–914. 10.1016/j.im.2016.03.006.

[CR129] Zhang, L., Van Iddekinge, C. H., Arnold, J. D., Roth, P. L., Lievens, F., Lanivich, S. E., & Jordan, S. L. (2020). What's on job seekers' social media sites? A content analysis and effects of structure on recruiter judgments and predictive validity. *Journal of Applied Psychology, 105*(12), 1530–1546. 10.1037/apl0000490. 32162953 10.1037/apl0000490

[CR130] Zide, J., Elman, B., & Shahani-Denning, C. (2014). LinkedIn and recruitment: How profiles differ across occupations. *Employee Relations, 36*(5), 583–604. 10.1108/ER-07-2013-0086.

